# Structural and Optical Characterization of Porous NiV_2_O_6_ Films Synthesized by Nebulizer Spray Pyrolysis for Photodetector Applications

**DOI:** 10.3390/mi15070839

**Published:** 2024-06-28

**Authors:** Ahmed Kotbi, Islam M. El Radaf, Ilham Hamdi Alaoui, Anna Cantaluppi, Andreas Zeinert, Abdelilah Lahmar

**Affiliations:** 1 Laboratory of Condensed Matter Physics, University of Picardie Jules Verne, 33 Rue Saint Leu, 80039 Amiens, France; ahmed.kotbi@u-picardie.fr (A.K.); ilham.hamdi.alaoui@u-picardie.fr (I.H.A.); anna.cantaluppi@u-picardie.fr (A.C.); andreas.zeinert@u-picardie.fr (A.Z.); 2 Electron Microscope and Thin Films Department, Physics Research Institute, National Research Centre, 33 El Bohoos Str., Dokki, Giza 12622, Egypt; elradaf11b@gmail.com; 3 Department of Physics, College of Science, Qassim University, Buraydah 51452, Saudi Arabia

**Keywords:** NiV_2_O_6_ thin films, porous microstructure, absorption coefficient, energy gap, photodetector

## Abstract

NiV_2_O_6_ thin films were grown on glass slides with varying thicknesses using nebulizer spray pyrolysis. The impact of thickness on the thin films’ optical, structural, morphological, and electrical characteristics was systematically investigated. X-ray diffraction and micro-Raman analysis confirmed the formation of the triclinic NiV_2_O_6_ system. Surface morphology and roughness variations in the as-deposited NiV_2_O_6_ films were studied using scanning electron microscopy (SEM) and a profilometer. Optical properties, including optical band gap (Eg), extinction coefficient (k), absorption coefficient (α), and refractive index (n), were determined through optical reflectance and transmittance measurements. The optical energy gap of the as-deposited NiV_2_O_6_ films decreased from 2.02 eV to 1.58 eV with increased layer thickness. Furthermore, the photo-detectivity of the films demonstrated an enhancement corresponding to the prolonged spray time. The sensitivity values obtained for visible irradiation were 328, 511, and 433 for samples S1, S2, and S3, respectively. The obtained results can be imputed to the specific porous microstructure.

## 1. Introduction

Recently, much attention has been paid to metal oxide thin films due to their remarkable and captivating optical, optoelectronic, and electrical characteristics. These metal oxides exhibit exceptional thermal stability and possess a significant absorption coefficient. The distinctive characteristics of these metal oxides render them well-suited for various applications, such as optical memory devices, window layers for solar cells, IR sensors, and photodetectors. Photodetection converts light into an electrical signal, which is crucial in many modern technologies [1].

Among the widely used materials for photodetection are semiconductors such as silicon [2], III-V compounds like gallium arsenide [3], and other materials based on nanomaterials, including graphene [4,5,6,7,8], 2D transition metal dichalcogenides (TMDCs) [9], and graphitic-carbon nitride (g-C_3_N_4_) [10,11]. Graphene was the initial 2D material explored for photodetectors, capitalizing on its remarkable electrical characteristics, notably its impressive carrier mobility [6,7] and a high-speed bandwidth of up to 40 GHz [5,6,7]. However, the absence of a bandgap in graphene results in dark currents, contributing significantly to signal noise. This limitation substantially restricts its broader applications in photodetection. Among TMDs, molybdenum disulfide (MoS_2_) has attracted much interest in the last decade due to its high mobility, high optical absorption capacity and tunable bandgap [12,13,14]. These materials have specific properties that make them suitable for various wavelength ranges and diverse applications, ranging from detecting low light levels to high-speed optical communication. Exploring the properties of these materials opens up exciting possibilities for enhancing photodetection performance and expanding its applications in areas such as remote sensing, medicine, and quantum technology.

The use of advanced materials in developing optoelectronic devices, such as photodetectors, has become crucial to meet increasing performance requirements. A new material, NiV_2_O_6_, a compound of nickel and vanadium, emerges as a promising candidate for photodetection applications due to its inimitable properties. NiV_2_O_6_ has intrinsic characteristics that make it an attractive material, including its tunable optoelectronic properties. The ease of large-scale manufacturing of NiV_2_O_6_, its wide band gap of 2.4 eV [15,16], sensitive optical responses, high absorption coefficient exceeding 10^4^ cm^−1^, and its porous structure make it an attractive material for various applications. The porous films produced through this method prove highly valuable, finding potential applications in areas requiring extensive specific surfaces, such as gas sensors [17], supercapacitors [18], and batteries [19]. Additionally, these highly porous substrates can lead to a significant funneling effect [20]. In general, such materials find their use in sound absorption, thermal insulation and electrical applications. However, it is important to mention that all applications of porous materials are linked to the characteristics of the pores. To a large extent, the properties of porous materials depend on porosity, pore morphology, and pore size and size distribution, which are the most fundamental intrinsic factors [21].

For instance, the light trapping technique is widely used as an appropriate way to improve the efficiency of solar cells generally using silicon bottom texturing or a transparent conductor in superstrate-type cells, compared to non-porous structures [22]. Further, high-efficiency photoelectrochemical water splitting was reported for porous TiO_2_, which was almost two times that of the photoanode formed by TiO_2_ nanoparticle-based films (P25) [23]. This paper deals with the fabrication of large-area porous materials using a simple and inexpensive technique. We have chosen photodetection as an application among several others to highlight the capabilities of our material. Also, it can be used for very high-efficiency multi-junction solar cells because of its light absorption and trapping characteristics [24].

In the present work, we emphasize the fundamental properties of porous NiV_2_O_6_ film, its synthesis process, and its potential as a light-sensitive material for photodetectors, providing insight into its possibilities in optical sensing.

## 2. Materials and Methods

This work used a spray pyrolysis process to fabricate NiV_2_O_6_ layers. The glass substrates were cleaned by subjecting them to sonication at a temperature of 60 °C in an ultrasonic bath. Acetone, isopropanol, and deionized water were used sequentially for a duration of 10 min each. Subsequently, the substrates were dried using air. The precursor solution was produced from the chemical reaction between two solutions. We added 40 mL of 0.1 M nickel nitrate Ni (NO3)_2_ (Sigma Aldrich (Burlington, VT, USA) 99.99%) to 60 mL of 0.2 M of ammonium vanadate NH_4_VO_3_ (Sigma Aldrich 99.99%). All of these chemicals were dissolved in ethanol. The mixture was stirred for 15 min at a temperature of 60 °C. The type of substrate used in this work is a soda lime glass substrate. The heated glass slides were sprayed with the NiV_2_O_6_ solution for 4, 6, and 9 min for the S1, S2 and S3 sample, respectively. The spray pyrolysis settings were adjusted to a substrate temperature of 250 °C, 5 mL/min for the flow rate, and 3 atm for the air pressure. The distance between the nozzle and the substrate was adjusted to 30 cm. Using the Bruker–Dektak stylus profiler, the thickness of the NiV_2_O_6_ layers was measured (S1 was 0.86 µm, S2 was 2.41 µm, and S3 was 2.94 µm). The spray system used in this work was the HOLMARK spray pyrolysis Model HO-TH-04. The structural analysis of the phase formation was detected by an X-ray diffraction (XRD)-type four-circle Bruker Discover D8 diffractometer with CuKα = 1.5406 Å (D8 Advance, Bruker, Germany). The vibrational analyses were recorded with a micro-Raman Renishaw spectrometer using a green laser excitation source (532 nm). The microstructure and the composition of the NiV_2_O_6_ films were investigated by the scanning electron microscope (SEM) Quanta 200 FEG equipped with energy-dispersive X-ray spectroscopy (EDX). The optical properties were analyzed using a JASCO V-670 UV–vis–near-IR spectrometer with a PIN-757 Horizontal sampling integrating sphere in transmittance and reflectance modes. The thickness and roughness of the films were determined using a Bruker profilometer (model: Dektak XT). The photo-response of the deposited samples was characterized under a 1.5 G AM spectrum of an Ossila solar simulator (model No. G2009A1) at a constant applied bias of 3V using a Palmsens 4 electrical measurements station using the two-probe method at room temperature.

## 3. Results and Discussion

### 3.1. Microstructural and Surface Morphology Study

**Figure 1:**
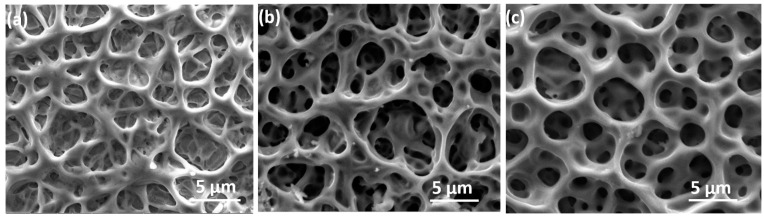
SEM images showing the microstructure of the investigated thin films: (**a**) S1 sample; (**b**) S2 sample and (**c**) S3 sample.

The microstructural characteristics of the prepared NiV_2_O_6_ films were examined using a scanning electron microscope, as shown in Figure 1. SEM images reveal complex and heterogeneous three-dimensional surfaces featuring irregularly dispersed pores of varying sizes and shapes. Image (a) of sample S1 exhibits the texture of a dense and porous structure with predominantly superficial pores. In contrast, images (b and c) of samples S2 and S3 depict deeper pores, resulting in significantly larger specific surface areas.

**Figure 2:**
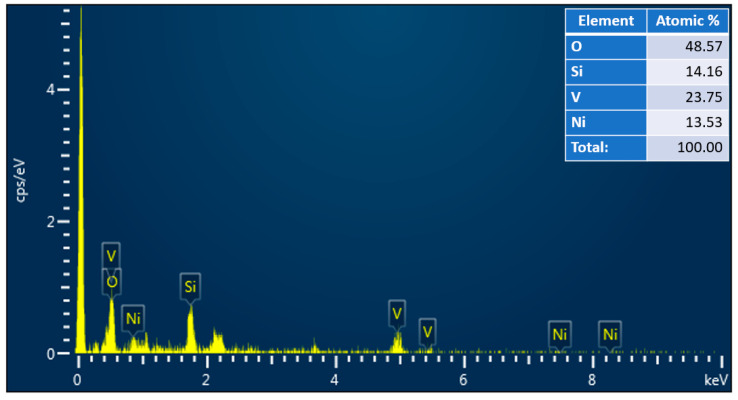
Example of EDX spectra with the atomic percentage of a NiV_2_O_6_ thin film.

The porous and meshing structure of NiV_2_O_6_ makes it an attractive material for various applications such as photodetection and gas sensors, which will benefit from a large specific surface area. Moreover, the EDX spectra of the elaborated samples are illustrated in Figure 2. The EDX spectra displayed the existence of Ni, V, and O peaks in all samples, and their atomic ratios are 1:2:6, respectively.

**Figure 3:**
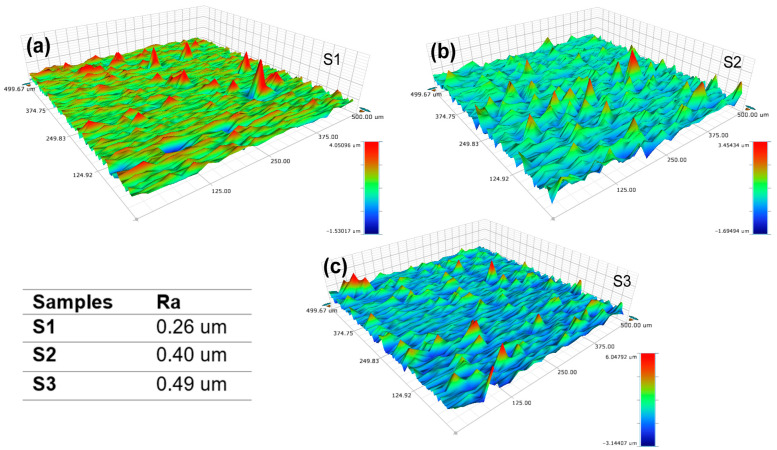
Profilometer mapping of thin layers of NiV_2_O_6:_ (**a**) S1 sample; (**b**) S2 sample and (**c**) S3 sample.

On the other hand, the roughness and surface morphology of the NiV_2_O_6_ films were assessed through mapping using a profilometer. Figure 3a–c present 3D micrographs of NiV_2_O_6_ thin films captured in a scanning area of (0.5 × 0.5) mm^2^. These images clearly illustrate the correlation between surface roughness and film thickness. It is noted that the surface roughness escalates as the film thickness increases. Specifically, the average film roughness increases from 0.26 µm to 0.49 µm as the film thickness increases. This trend will increase the quantity of trapped light and, therefore, the light–material reaction, i.e., the absorption for the very thick samples S2 and S3.

### 3.2. Structural Investigation

**Figure 4:**
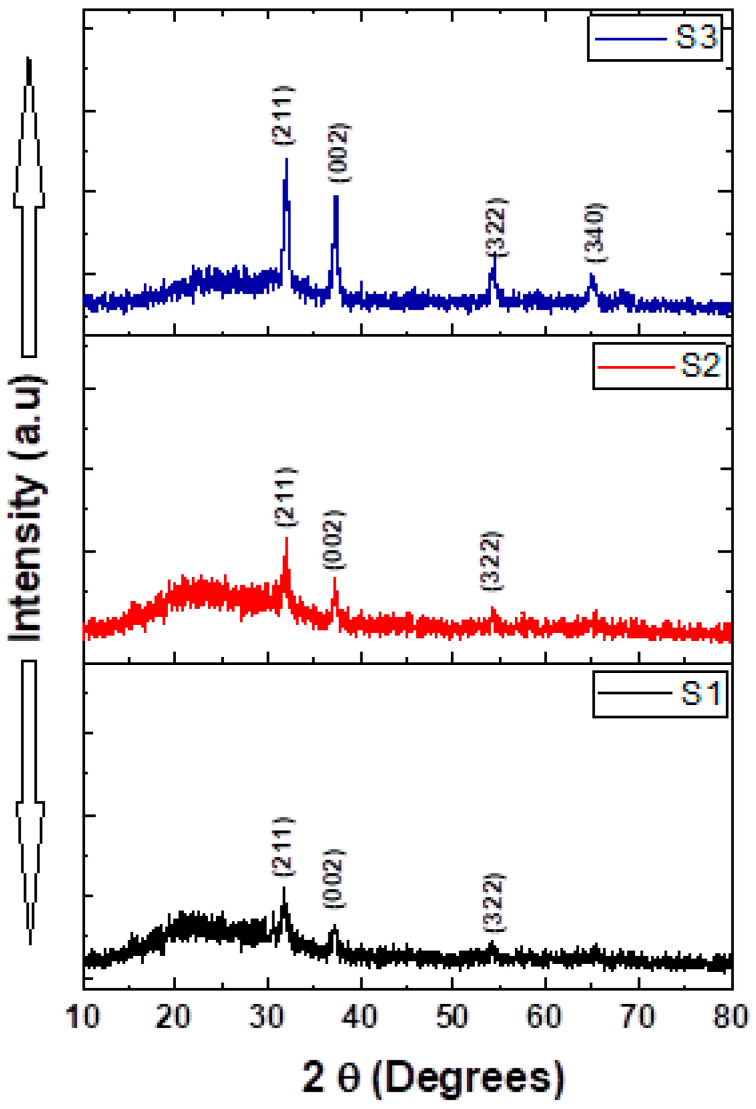
X-ray diffraction patterns of the porous NiV_2_O_6_ films.

Figure 4 depicts the XRD of the NiV_2_O_6_ thin films, in the limit of a detection device. The figure displays only four distinguished diffraction patterns. According to the Standard JCPDS file no. 76-0359, the observed peaks might be indexed with (211), (002), (322), and (340) planes of the NiV_2_O_6_ single triclinic structure.

It is agreed upon that at a constant temperature, the extension of the time (herein the prolonged spray time) could influence the degree of migration and rotation of grain boundaries, and especially, the degree of grain recrystallization becomes more complete. We believe that this was the case for S3, as there is a clear difference in peak widths compared to the S1 and S2 samples. Complementary structural information is needed to confirm this assumption.

**Figure 5:**
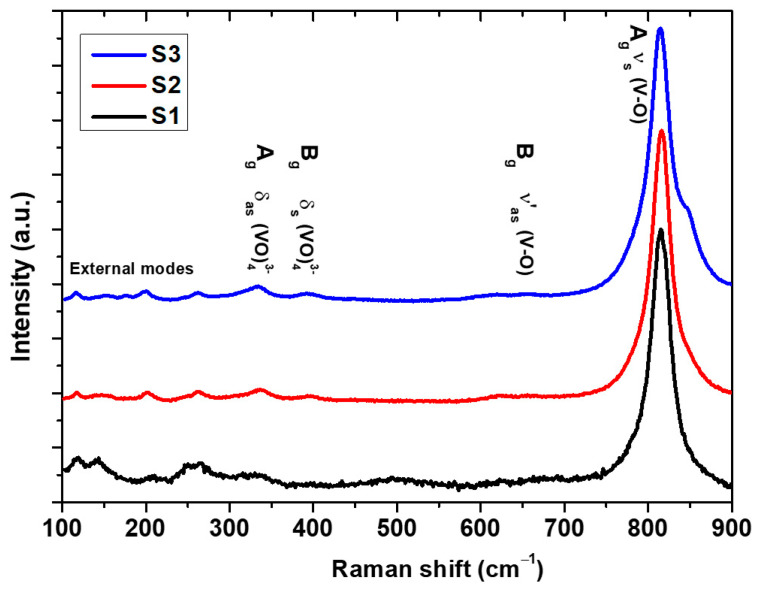
Raman spectra of the investigated NiV_2_O_6_ films.

Raman spectroscopy is an effective tool for investigating structural and bonding characteristics, providing valuable insights into materials’ local structure, crystallization and electronic properties. The Raman bands of NiV_2_O_6_ in the 100–900 cm^−1^ range were obtained with varying spray times, as depicted in Figure 5.

Consistent Raman bands at 327, 367, 647, and 816 cm^–1^ were observed across all samples, indicative of the distinctive vibrational bands of the triclinic NiV_2_O_6_ system. The comparison of vibration modes for different materials like NiV_2_O_6_ is summarized in Table 1. The prominent band at 816 cm^−1^ corresponds to the shorter symmetric V–O stretching mode (Ag), while the weaker band at 647 cm^−1^ is associated with the short (Bg) asymmetric V–O stretching modes [25]. Additionally, the asymmetric and symmetric bending vibrations of the VO_4_ tetrahedron were identified at 327 and 367 cm^−1^, respectively [26]. Alternatively, within our sample S3, we detected a shoulder at 846 cm^−1^, which can be ascribed to a division arising from the lower symmetry of the monoclinic structure [27]. The vibrations of the crystal lattice (external modes) are responsible for the peaks observed at 138 and 205 cm^−1^ [28].

### 3.3. Optical Study

**Figure 6:**
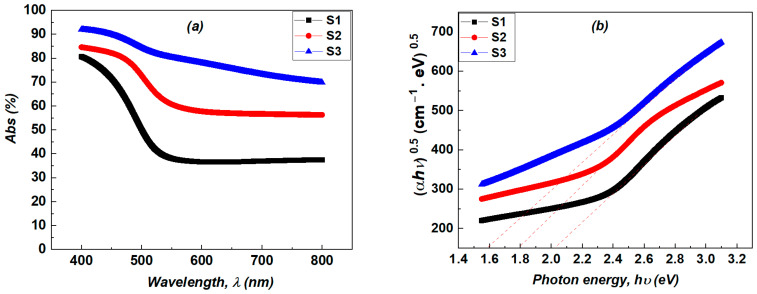
(**a**) Absorptance spectra of NiV_2_O_6_ films synthesized at different thicknesses. (**b**) A Tauc plot for a NiV_2_O_6_ fabricated thin film.

The absorption coefficient (α) was determined using the following formula [31]:

(1) $$\alpha =\frac{1}{d}ln\left[\frac{{\left(1-R\right)}^{2}}{2T}+{\left(\frac{{\left(1-R\right)}^{4}}{{4T}^{2}}+R^{2}\right)}^{1/2}\right]$$

where *d* represents the film thickness, *T* signifies transmittance, and *R* is the reflectance of the samples. The optical bandgap energy (*E_g_*) was derived from the absorption coefficient (α) through the application of the Tauc model [32,33] shown below:

(2) $$\alpha h\vartheta =A(h\vartheta -E_{g}{)}^{m}$$

where hϑ represents the photon energy, *A* is a constant, *E_g_* is the optical band gap, and *m* is an exponent indicating the nature of optical absorption. For a direct authorized transition, *m* = 1/2; for a direct authorized transition, *m* = 2; for an allowed indirect transition, *m* = 3/2; and for a direct forbidden transition, *m* = 3. The bandgap energy (*E_g_*) of the developed NiV_2_O_6_ was obtained by plotting (*αhν*)^1/2^ versus photon energy considering the indirect band gap reported for this material [34] (Figure 6b).

The dielectric function $\varepsilon \left(\omega \right)$ is a critical factor for the optical properties of a semiconductor, which could be defined by the below equation [35,36]:

(3) $$\varepsilon \left(\omega \right)=\varepsilon_{1}\left(\omega \right)-i\varepsilon_{2}(\omega )$$

where $\varepsilon_{1}\left(\omega \right)$ is the real part of the dielectric function, and $\varepsilon_{2}(\omega )$ is the imaginary part of the dielectric functions, which can represent electronic transitions linked to conduction and valence bands.

**Figure 7:**
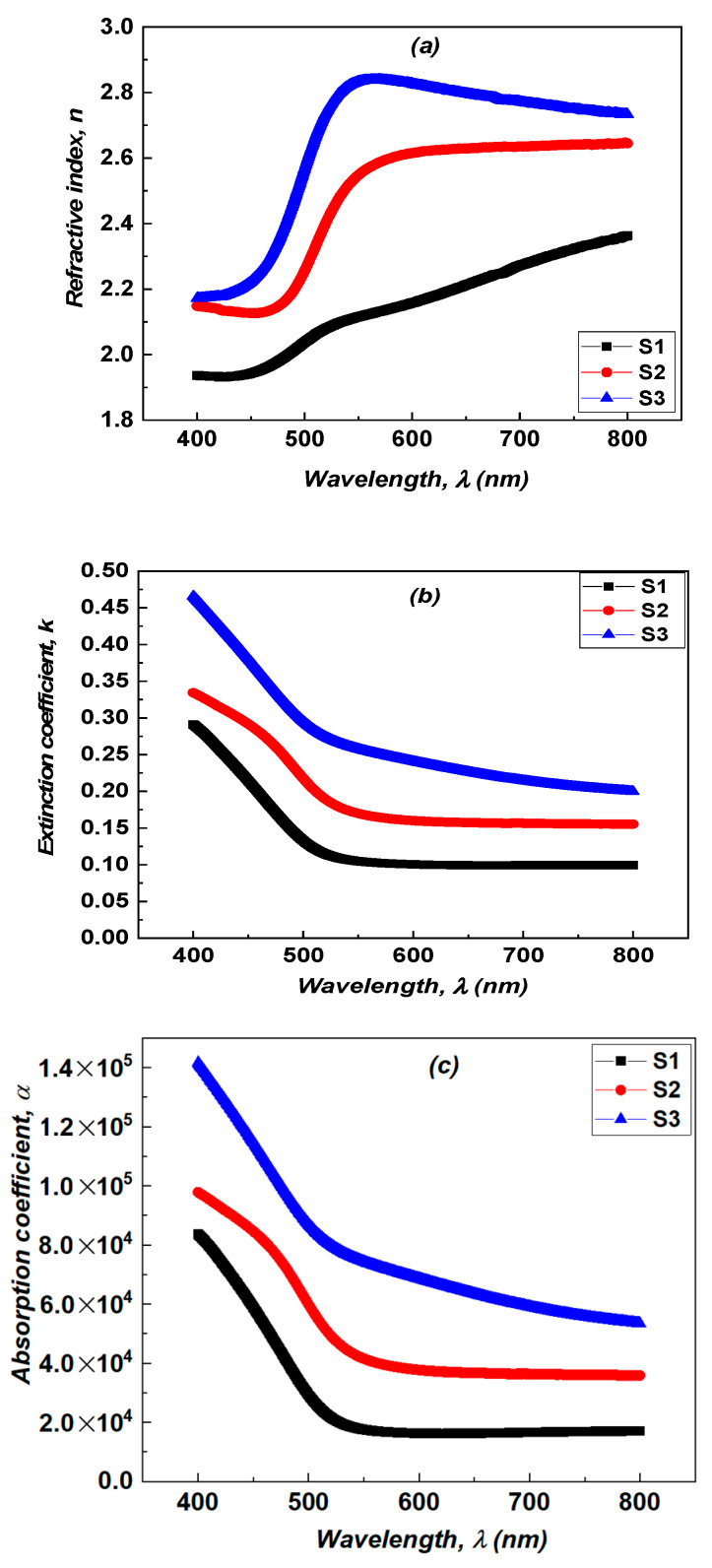
(**a**) The variation in n of the NiV_2_O_6_ films versus λ. (**b**) The extinction coefficient of the NiV_2_O_6_ films versus λ and (**c**) the absorption coefficient of the NiV_2_O_6_ films versus λ.

The refractive index is a characteristic of a medium, describing the behavior of light in it, whose complex form $n^{*}=n-ik$ [30,31], where *n* represents the refractive index, and *k* refers to the extinction coefficient calculated by the following equation *k* = *αλ*/4*π* . The variations of the absorption coefficient α, the extinction coefficient k and the refractive index *n* as a function of wavelength are given in Figure 7a–c. These parameters inform us about the loss of energy in the medium, i.e., the absorption of the photons. NiV_2_O_6_ is a promising material for fabricating photodetector devices due to its suitable optical bandgap energy of 2.02 eV, and its high optical absorption coefficient > 10^4^ cm^−1^ [16,32]. It is important to emphasize that the porous structure of our samples leads to optical data relative to this effective medium (NiV_2_O_6_ + the pores). These data do not uniquely refer to the NiV_2_O_6_ phase of the spongy material. It is well known that NiV_2_O_6_ is a semi-conductor and one therefore expects very low absorption for energies below the bandgap, in contrast to what can be observed in our samples where the absorbed energy is higher than 40% for the longer wavelengths. This is owed to the highly porous structure of the material. As sample S1 exhibits a higher density and a less spongy structure than the other two samples, its band gap is near to that expected for NiV_2_SO_6_, while for S2 and S3, the lower bandgaps reflect their more porous microstructure. This is underlined by the very high energy fraction absorbed even at higher wavelengths for these samples. Therefore, this relative decrease in *E_g_* does not mean that the electronic structure of the NiV_2_SO_6_ phase has changed (there is no quantum confinement for instance) for S2 and in S3.

On the other hand, the method of development and the way of processing our material allowed us to obtain a sponge-like material that has high porosity, which helps the material to absorb even at sub-gap energies. This is the strong point of our material, which will certainly be advantageous for photovoltaic, photocatalytic and other applications.

### 3.4. Photodetector Application

**Figure 8:**
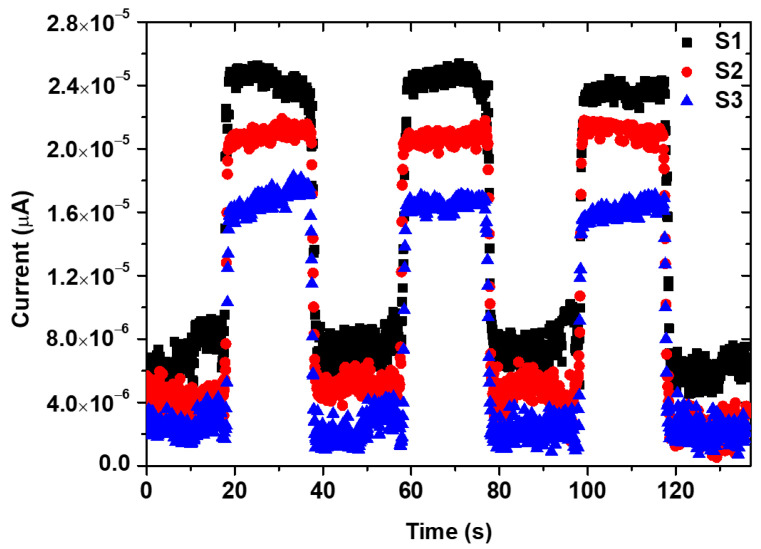
J-t characteristics of NiV_2_O_6_ films below chopped AM 1.5 (100 mW·cm^−2^) lighting.

In this work, sensitivity was studied with different film thicknesses at a constant light source intensity (100 mW/cm^2^) and effective illumination area (0.1 cm^2^) (Figure 8). The sensitivity depends on the intensity of the light source and the effective illumination area. The photodetector’s performance was evaluated by a key parameter known as sensitivity (*S*), which can be defined as follows [37]:

(4) $$S\left(\%\right)=\frac{I_{ph}}{I_{dark}}\times 100$$

where *I_ph_* is the photocurrent, and it is equal to *I_light_* − *I_dark_*; moreover, *I_dark_* is the dark current, and *I_light_* is the light current. At a bias voltage of 3 V, the sensitivity values for samples S1, S2, and S3 were computed and found to be around 328, 511, and 433, respectively, as presented in Table 2. It was observed that the sensitivity of the layers increased with the boost in layer thickness due to the increase in the optical absorption of the films.

**Figure 9:**
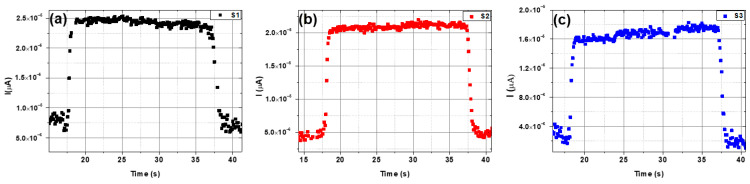
Rise and decay time under 3 V bias voltage for the investigated samples (**a**) S1, (**b**) S2 and (**c**) S3.

Rise and decay times are crucial parameters in assessing photodetector performance. For a single on/off cycle, the rise times were determined to be approximately 0.62, 0.29, and 0.43 s. In contrast, decay times were found to be 1.19, 0.52, and 0.69 s at a bias voltage of 3 V for samples S1, S2, and S3, respectively (Figure 9). Sample S2 exhibited very fast rise and decay times, as revealed by the photo-response time results.

## 4. Conclusions

This research involved the successful fabrication of NiV_2_O_6_ thin films on glass substrates through the application of the spray pyrolysis technique. The influence of spray time on the optical properties of NiV_2_O_6_ films was investigated. A triclinic structure was obtained for all samples. The reflectance and transmission spectra were utilized to compute the optical constants, including the refractive index (n) and extinction coefficient (k). With increased film thickness, the effective band gap value decreased from 2.02 eV to 1.58 eV. The film’s photo response parameters, like sensitivity (S), increased with film thickness. The fastest rise and fall times of 0.29 s and 0.52 s were obtained for sample S2.

However, it is interesting to point out that the properties obtained are not intrinsic to the films alone. The specific microstructure in which the pores take place, and change depth with increasing thickness, plays a key role in trapping light and changing the behavior that would be observed if the microstructure of the films were continuous and smooth. The versatile method used herein for processing films with large active areas is promising for a wide range of applications.

The study performed in this paper successfully demonstrates that large-area porous materials can be fabricated using a simple and inexpensive technique (spray pyrolysis). Application of these materials to photodetection reveals their sensing potential. Moreover, the unique light absorption and trapping characteristics of the porous materials suggest that they are suitable for integration into high-efficiency multijunction solar cells. These results indicate that the fabricated materials not only meet initial expectations but also open new avenues for advances in various optoelectronic applications.

Evidently, the characteristics of the pores in terms of fill factor, size and spatial distribution determine the properties of these materials.

Further research to understand this relationship is needed, particularly through modeling and simulation, not only to tailor materials to a specific application, but also to control their preparation.

## Figures and Tables

**Table 1:** Vibrational modes observed in Raman spectrum for different materials like NiV_2_O_6_.

Materials	Assignment	Raman Shift (cm^−1^)
CoV_2_O_6_	_S_V-O-V	775 [25]
	_as_V-O	851 [25]
	_as_V-O	913 [25]
	_S_V-O	946 [25]
Li_0.8_NiVO_4_	δ_as_ (VO_4_)	332 [29]
	ν_as_ (V-O)	795 [29]
	ν_s_ (V-O)	825 [29]
Cu–BiVO_4_	External modes	210 [27]
	δ_as_ (VO_4_)	327 [27]
	δ_s_ (VO_4_)	367 [27]
	ν′_as_ (V-O)	637 [27]
	ν_as_ (V-O)	710 [27]
	ν_s_ (V-O)	819 [27]
BiVO_4_	δ_as_ (VO_4_)	324 [26]
	δ_s_ (VO_4_)	367 [26]
	ν_as_ (V-O)	710 [26]
	ν_s_ (V-O)	811 [26]
Mo–BiVO_4_	External modes	213 [30]
	δ_as_ (VO_4_)	327 [30]
	δ_s_ (VO_4_)	367 [30]
	ν′_as_ (V-O)	642 [30]
	ν_as_ (V-O)	710 [30]
	ν_s_ (V-O)	831 [30]
BiVO_4_	External modes	27, 211 [28]
	δ_as_ (VO_4_)	324 [28]
	δ_s_ (VO_4_)	368 [28]
	ν_as_ (V-O)	703 [28]
	ν_s_ (V-O)	828 [28]
NiV_2_O_6_	External modes	138, 205 (This work)
	δ_as_ (VO_4_)	327 (This work)
	δ_s_ (VO_4_)	367 (This work)
	ν′_as_ (V-O)	647 (This work)
	ν_s_ (V-O)	816 (This work)

**Table 2:** Photodetector performance compared with those reported by others.

Samples	Rise Time (s)	Decay Time (s)	Sensitivity (%)	Ref.
S1	0.62	1.19	328	This work
S2	0.29	0.52	511	This work
S3	0.43	0.69	433	This work
Fe-doped ZnO/BiVO_4_	0.17	-	2900	[38]
SnS	0.44	0.50	697	[39]
GaTe	0.045	0.045	788	[40]
MoSe_2_	0.4	0.2	1626	[41]

## Data Availability

The original contributions presented in the study are included in the article, further inquiries can be directed to the corresponding author.
